# Multimodal ultrasound: a non-invasive method for identifying dedifferentiation of papillary thyroid carcinoma during active surveillance

**DOI:** 10.3389/fonc.2025.1545407

**Published:** 2025-02-19

**Authors:** Qian-Yi Dou, Huan-Ling Guo, Wan-Bing Qiu, Ming Xu, Shu-Ling Chen, Xiao-Er Zhang, Xiao-Yan Xie, Jin-Yu Liang

**Affiliations:** ^1^ Department of Medical Ultrasonics, Institute of Diagnostic and Interventional Ultrasound, The First Affiliated Hospital of Sun Yat-Sen University, Guangzhou, China; ^2^ Department of Medical Ultrasonics, Shenzhen People’s Hospital, Shenzhen, Guangdong, China

**Keywords:** papillary thyroid carcinoma, anaplastic thyroid carcinoma, multimodal ultrasound, active surveillance, aggressiveness

## Abstract

**Objectives:**

To assess the diagnostic accuracy of multimodal ultrasound in differentiating anaplastic thyroid carcinoma (ATC) from papillary thyroid carcinoma (PTC) and to evaluate its capability in detecting thyroid carcinoma (TC) aggressiveness.

**Methods:**

Sixty nude mice were randomly assigned to ATC and PTC groups and injected subcutaneously with KHM-5M and TPC-1 cell lines, respectively. Tumors were analyzed using B-mode ultrasound (B-US), Color Doppler flow imaging (CDFI), elastography, and contrast-enhanced ultrasound (CEUS). A logistic model integrating multimodal ultrasound data was constructed, and Ki-67 and CD31 expressions in tumor tissues were analyzed immunohistochemically. Correlations between ultrasound features and aggressiveness markers were investigated.

**Results:**

The ATC group exhibited significantly higher strain-elastography scores (*p*=0.009) and Adler grades in CDFI (*p*=0.045). CEUS revealed a higher frequency of heterogeneous enhancement (95.2% vs. 48.1%, *p*<0.001) and perfusion defects (90.5% vs. 63.0%, *p*<0.001) in ATC. Model area under the curve (AUC) values for distinguishing ATC from PTC were 0.963 for (B-US + CEUS), 0.926 for CEUS, 0.729 for elastography, 0.663 for CDFI, and 0.675 for B-US. The multimodal ultrasound model demonstrated significant correlations with Ki-67 (*p*<0.001) and microvessel density (MVD) (*p*<0.001).

**Conclusions:**

Multimodal ultrasound showing high efficacy with an AUC of 0.963 for B-US and CEUS combined in distinguishing ATC from PTC and exhibited strong associations with Ki-67 and MVD. Incorporating multimodal ultrasound, with an emphasis on CEUS, into active surveillance strategies for PTC is recommended. By providing detailed insights into tumor vascularity and aggressiveness, multimodal ultrasound could play a crucial role in early detection and treatment decision-making, improving patient outcomes.

## 1 Introduction

Papillary thyroid carcinoma (PTC), accounting for over 80% of thyroid carcinoma (TC) cases (1), is a well-differentiated cancer type, with a cure rate exceeding 90% (2). Owing to the indolent nature of low-risk PTC (≤ 1 cm), active surveillance (AS) has been globally adopted as an alternative to immediate surgery (3, 4). However, the condition in approximately 2.9–9.7% of papillary thyroid microcarcinoma cases has shown progression during AS (5). Moreover, long-term untreated differentiated PTC may undergo dedifferentiation—a biological process transforming cancer from a highly differentiated state to a poorly differentiated one (6), which leads to progression to anaplastic thyroid carcinoma (ATC) (7, 8). ATC, a rare but lethal type of TC, is characterized by rapid local growth and early distant metastasis (9). The median overall survival for typical ATC ranges from 4 to 12 months, with the treatment being limited to palliative or supportive care in most patients (10, 11). These findings indicate a critical need to develop methods to detect ATC early during AS for PTC.

During AS for PTC, disease progression—marked by tumor enlargement, new lymph node metastasis (LNM), extrathyroidal extension (ETE), or distant metastasis—is primarily assessed using B-mode ultrasound (B-US) (12). While B-US effectively distinguishes benign from malignant thyroid nodules, its capability to differentiate pathological types is limited. Advancements in multimodal ultrasound, including contrast-enhanced ultrasound (CEUS) and elastography (strain-elastography [SE] and shear wave elastography [SWE]), have shown promise in providing tumor typing insights. CEUS enhances visualization of lesion vascularity, whereas elastography assesses tissue elasticity changes owing to specific pathological or physiological processes. For instance, Du et al. (13) highlighted the role of multimodal CEUS in distinguishing small cell lung cancer from non-small cell lung cancer, emphasizing that vascular patterns observed via CEUS-based micro-blood flow imaging provide evidence for the differential diagnosis of peripheral pulmonary tumors. Similarly, Guo et al. (14) demonstrated significant differences in SWE’s maximal elasticity among various pathological types of malignant liver lesions. Studies (15) have reported that, compared to B-US alone, combining B-US, CEUS, and SWE improves the differential diagnosis of thyroid nodules smaller than 10 mm. However, the role of multimodal ultrasound in tumor typing or identifying dedifferentiated components remains unexplored.

Therefore, this study aimed to evaluate the diagnostic performance of multimodal ultrasound in differentiating ATC from PTC. Additionally, as tumor cell dedifferentiation correlates with increased aggressiveness (16, 17), the secondary objective was to investigate the relationship between multimodal ultrasound features and markers of TC aggressiveness, namely Ki-67 and microvessel density (MVD).

## 2 Materials and methods

### 2.1 Subcutaneous xenograft TC tumor in nude mice

Ethical approval was obtained from the Research Ethics Committee of Sun Yat-sen University (approval no. SYSU-IACUC-2024-000678). Female BALB/c nude mice aged 5–8 weeks were used in all experiments (Laboratory Animal Center, Sun Yat-Sen University). The human ATC cell line KHM-5M was provided by Professor Hai-Peng Xiao, and the PTC cell line TPC-1 was provided by Professor Jie Li. KHM-5M cells were cultured in Roswell Park Memorial Institute 1640 (RPMI 1640) medium and TPC-1 cells were cultured in Dulbecco’s Modified Eagle’s Medium (DMEM), respectively, with 10% fetal bovine serum and 1% penicillin/streptomycin at 37°C in a 5% CO_2_ incubator.

After acclimation for one week, the mice were randomly categorized into the ATC and PTC groups and inoculated subcutaneously with KHM-5M (2×10^6^ cells/mouse) and TPC-1 (1×10^7^ cells/mouse) in the right back, respectively (n=30). Tumor sizes were monitored every three days, and tumor volumes were calculated using the following formula: tumor size = (length × width^2^)/2. To minimize size-related imaging interference and ensure consistent examination timing, ultrasound examinations were performed when the mean tumor size reached 250 mm³ (the mean volumes of ATC and PTC groups reaching 250 mm³ in 15 and 37 days, respectively).

### 2.2 Multimodal ultrasound image acquisition

All the ultrasound examinations were performed by the same sonographer with > 10 years of experience in ultrasonography of TC. Ultrasonographic examinations were performed using the Aplio i900 (Canon, Tokyo, Japan) ultrasound equipment with built-in specialized analysis software for time-intensity curve (TIC), CEUS-matching imaging techniques, and a linear transducer with a 5.0–10.0 MHz frequency range. Ultrasound imaging for all the mice was performed under continuous anesthesia with 2% isoflurane. The acoustic coupling pad, placed over the skin region above the subcutaneous tumor, ensured optimal acoustic coupling between the probe and the skin. The ultrasound signal gain was set to 75%–90%, and the image depth was set to 2–3.5 cm. The focus point was maintained as close as possible to the lowest base of tumors. B-US was performed at the maximum cut surface of the tumor. The cut surfaces of the color Doppler flow imaging (CDFI), elastography, and CEUS examinations were kept consistent with those of B-US.

For SE, vertical light pressure was applied to the tumor, which was then decompressed to obtain an SE image. For SWE, the lesion of interest was placed at the center of the ultrasound image. The Q-box region was placed in the stiffest area. The experiment was repeated three times.

Subsequently, CEUS imaging was performed. An intravenous bolus of 0.1 mL of SonoVue (Bracco, Milan, Italy) was administered over 2 s, followed by a bolus of 0.1 mL of saline for another 2 s through the caudal vein. The recording started immediately at the time of injection. Each mouse was continuously scanned for at least 2 min. All the examinations were stored digitally.

### 2.3 Analysis of multimodal ultrasound image

Ultrasound images were analyzed by two sonographers who were blinded to the grouping (H.L.G., with 10 years of experience in ultrasound examination, and Q.Y.D., with three years of experience in ultrasound imaging). B-US features of the TC were assessed, including echogenicity (hyperechoic, isoechoic, or hypoechoic, as compared with those of the nearby muscle), edges (smooth or non-smooth), boundary (clear or unclear), and calcification (microcalcification or non-microcalcification). Blood flow richness was analyzed using the Adeler classification method (18): Grade 0 (no blood supply), Grade 1 (low blood supply), Grade 2 (medium blood supply), and Grade 3 (rich blood supply).

The identification of strain elasticity images was green for soft and red for hard and graded according to the following semi-quantitative assessment (19): score of 1, when the tumors and surrounding tissues are green; 2, when the tumors are mainly green; 3, when the tumors are primarily green, with surrounding tissues visible in red; 4, when the tumors are primarily red; and 5, when the tumors and surrounding tissues are all red. The mean stiffness value (E-mean) was automatically displayed using ultrasound SWE software. SWE results of SWE were presented as the average of three repeated experiments.

The primary features of CEUS were observed, including the homogeneity of enhancement (homogeneous or heterogeneous), direction of enhancement (centripetal, centrifugal, or diffuse), and perfusion defects (present or absent). Quantitative analysis of tumor enhancement was conducted using the ultrasound equipment in the TIC analysis software. The regions of interest (ROI) included the entire tumor, tumor parenchyma (excluding necrotic areas), and the muscle next to the tumor (**Supplementary Figure 1**). The following characteristics of the TICs were analyzed (20, 21): Peak intensity (PI) was defined as the maximum intensity of the time-intensity curve. Time to peak (TTP) was defined as the time needed to reach PI beginning from the time the first microbubble reached the lesion. Mean transit time (MTT) was defined as the time for contrast media to pass through the ROI. Wash-in slope (slope) was defined as the maximum wash-in velocity of the contrast. Area under the curve (AUC) was proportionate to the total volume of blood in the ROI, including wash-in area under the curve (WiAUC) and wash-out area under the curve (WoAUC).

### 2.4 Immunohistochemistry

All tumor samples were collected immediately after multimodal ultrasound examination. Tumors were sectioned along the largest long-axis cross section, imaged, and subsequently preserved in 4% formalin until IHC was performed to ensure that the pathology analysis slice was consistent with the imaging section. For IHC, paraffin sections were dewaxed and subjected to antigen retrieval. Deparaffinized tissue sections were incubated with 3% hydrogen peroxide for 10 min and blocked with bovine albumin for 30 min. Next, the sections were incubated overnight at 4°C with anti-CD31 antibody (1:1000, Servicebio, GB113151) and anti-Ki-67 antibody (1:1000; Servicebio, GB111499), respectively. After washing, the tissue sections were incubated with horseradish peroxidase-conjugated secondary antibody (1:200, Servicebio, GB23303) at 37°C for 50 min. The sections were then stained with diaminobenzidine solution and counterstained with hematoxylin. Slides were scanned using a KFBIO KF-PRO-020 digital pathology slide scanner and analyzed using the KF-viewer software.

Ki-67 expression intensity was quantified as the percentage of Ki-67 positive nuclei (22). Yellow or dark brown nuclei indicated Ki-67 positivity. The number of Ki-67 positive cells per 100 tumor cells was counted under a microscope using a high-magnification field of view and expressed as a percentage. Randomly select 10 fields for each slide, and the average percentage of Ki-67 positive cells for the 10 fields.

Microvessel counting (CD31 count) was performed using Weidner’s method (23). Specifically, the entire slide was scanned at low magnification to find the area with the highest MVD and then switched to high magnification to count the number of microvasculatures. MVD was expressed as the average number of 5 random fields counted at a 20× objective magnification.

### 2.5 Statistical analysis

SPSS 25.0 (IBM Corporation, Armonk, NY, USA) was used for statistical analysis. Continuous variables were expressed as the mean ± standard deviation (SD). Normally distributed data were analyzed using the independent-sample *t*-test, whereas the Mann-Whitney *U* test was performed for the skewed distributed data. Binary variables were compared using the *χ^2^*-test and ordinal categorical variables were compared using the rank-sum test. Spearman’s correlation analysis was used to determine the correlation between multimodal ultrasonography and immunohistochemical index counts. A multimodal ultrasound model for distinguishing ATC from PTC was constructed by univariate and multivariate logistic regression. Variables with p<0.05 in the univariate analysis were used in the multivariate analysis, and the significant variables (p<0.05) in the multivariate analysis were then used to construct a risk prediction model (24, 25). Diagnostic efficiency was evaluated using an area under the receiver operating characteristic curve (AUC), sensitivity (SEN), specificity (SPE), accuracy (ACC), positive predictive value (PPV), and negative predictive value (NPV). The inter-observer agreement for subjective image reading was analyzed with the kappa test (κ < 0.40: poor agreement; 0.40 ≤ κ < 0.75: good agreement; and κ ≥ 0.75: excellent agreement). Statistical significance was set at *p*<0.05.

## 3 Results

### 3.1 Analysis of B-US, CDFI and elastography

**Figure 1:**
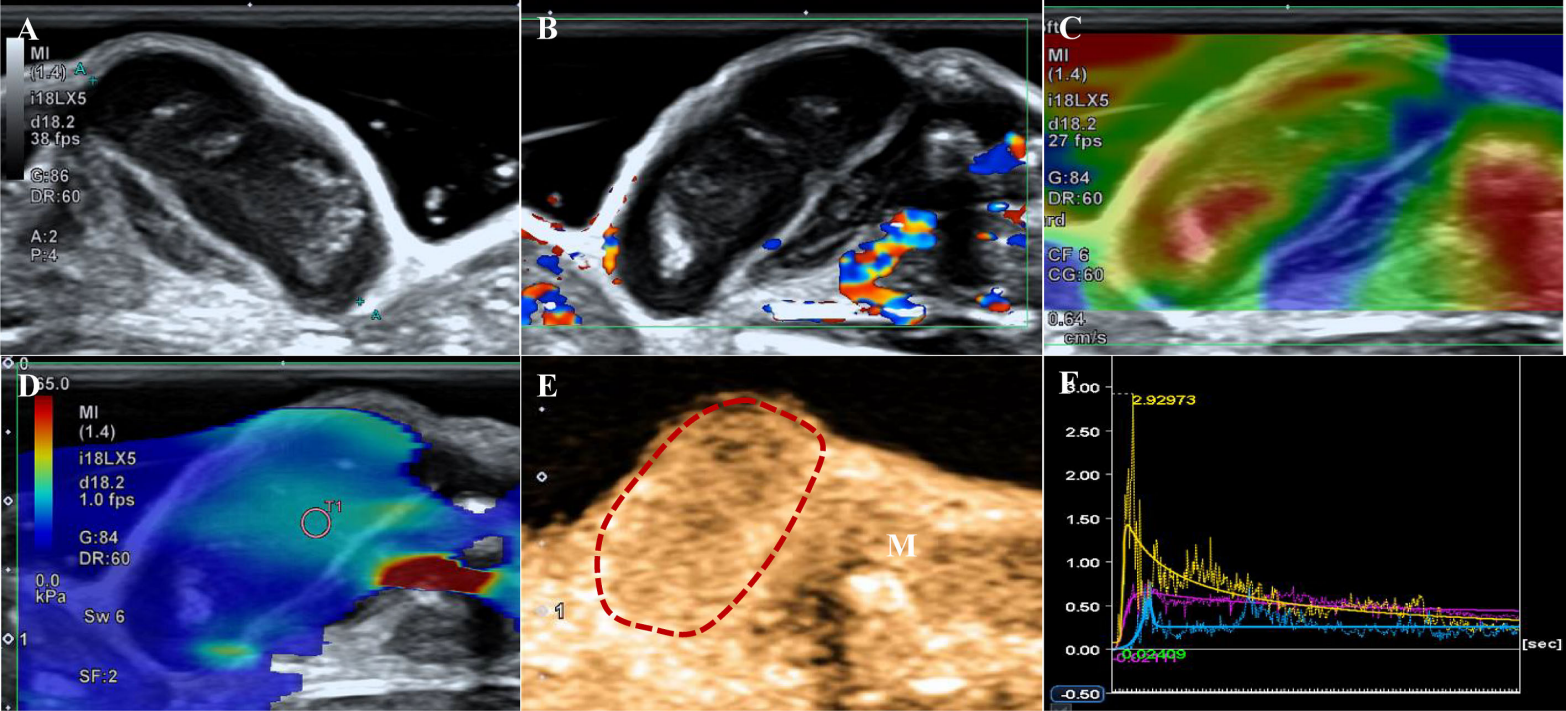
A tumor from the PTC group. **(A)** B-US shows a hypoechoic mass with a clear boundary and smooth edge, and punctiform microcalcification is observed. **(B)** CDFI image indicates Adler grade 1. **(C)** The strain elastography image shows strain-elasticity score 3. **(D)** The mean of the stiffest area of the tumor on the elasticity mode is 35.65 kPa. **(E)** CEUS shows a homogeneous enhancement image obtained 6 s after contrast agent injection (the dotted line represents the contour of the tumor, M indicates muscle). **(F)** Time-intensity curve. Purple: the entire tumor. Yellow: tumor parenchyma. Blue: the muscle next to the tumor.

**Figure 2:**
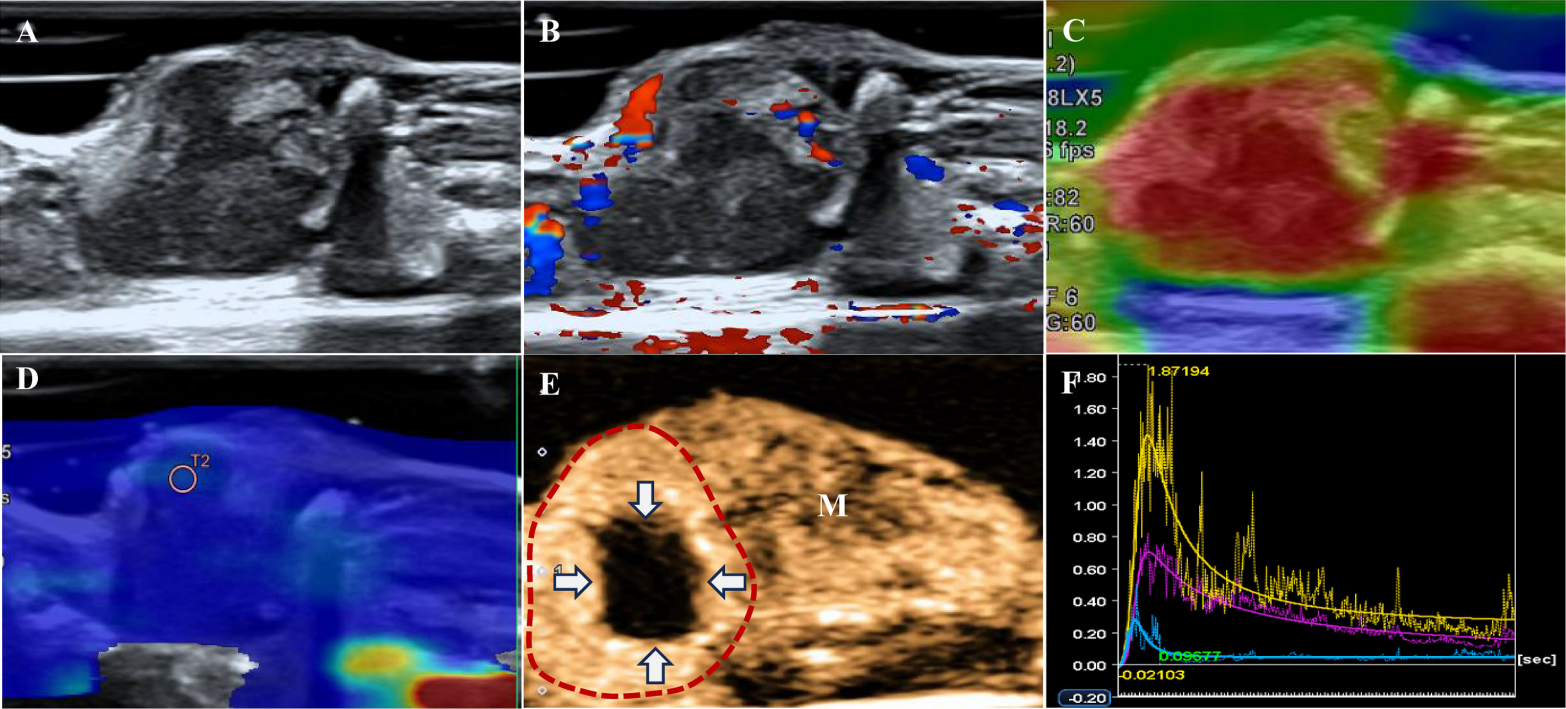
A tumor of the ATC group. **(A)** B-US shows an isoechoic mass with a clear boundary and non-smooth edge. **(B)** CDFI indicates Adler grade 3. **(C)** The strain elastography shows strain-elasticity score 5. **(D)** The mean of the stiffest area of the tumor on the elasticity mode is 18.75 kPa. **(E)** CEUS shows a local perfusion defect at 6s after contrast agent injection (the dotted line represents the contour of the tumor, white arrows represent a perfusion defect, M indicates muscle). **(F)** Time-intensity curve. Purple: the entire tumor. Yellow: tumor parenchyma (excluding necrotic areas). Blue: the muscle next to the tumor.

The inter-observer agreement (kappa statistic) ranged from 0.736 to 0.818. The B-US, CDFI, and elastography results are shown in **Table 1**. A total of 57.1% (12/21) of ATCs presented as isoechoic masses and 77.8% (21/27) of PTCs presented as hypoechoic masses (*p*=0.013). No significant differences were noted in the tumor volumes calculated based on the ultrasound measurement data (*p*>0.05). Among them, 16 (59.3%) PTCs and four (19.0%) ATCs showed microcalcifications (*p*=0.005, **Figures 1A**, **2A**). The tumors did not differ significantly in terms of boundaries (clear or not) or edges (smooth or not) between the two groups (*p*>0.05). The Adler grades of the CDFI differed significantly among the different pathological types of TC (*p*=0.045). The proportions of patients with Adler Grades 1, 2, and 3 in the ATC group were 57.1% (12/21), 19.1% (4/21), and 23.8% (5/21), respectively, whereas those in the PTC group were 81.5% (22/27), 14.8% (4/27), and 3.7% (1/27), respectively (**Figures 1B**, **2B**).

**Table 1:** Comparison of characteristics on multimodal ultrasound between PTC and ATC groups.

Characteristic	PTC (n = 27)	ATC (n = 21)	*P* value
Volume	259.40 ± 126.64	257.54 ± 207.32	0.406
Echogenicity			0.013*
Hyperechoic	0(0%)	0(0%)	
Isoechoic	6(22.2%)	12(57.1%)	
Hypoechoic	21(77.8%)	9(42.9%)	
Boundary			0.186
Clear	27(100%)	19(90.5%)	
Unclear	0(0%)	2(9.5%)	
Edge			0.342
Smooth	19(70.4%)	12 (57.1%)	
Non-smooth	8(29.6%)	9(42.9%)	
Calcification			0.005**
Non-microcalcification	11(40.7%)	17(81.0%)	
Microcalcification	16(59.3%)	4(19.0%)	
Adler grade			0.045*
Grade 0	0(0.0%)	0(0.0%)	
Grade 1	22(81.5%)	12(57.1%)	
Grade 2	4(14.8%)	4(19.1%)	
Grade 3	1(3.7%)	5(23.8%)	
SWE			0.959
Emean	32.14 ± 15.3	36.80 ± 27.88	
Strain-elasticity score			0.009**
Score 1	3(11.1%)	0(0.0%)	
Score 2	7(25.9%)	2(9.5%)	
Score 3	8(29.6%)	5(23.8%)	
Score 4	9(33.3%)	12(57.1%)	
Score 5	0(0.0%)	2(9.5%)	
Direction of enhancement			0.367
Centripetal	20(74.1%)	13(61.9%)	
Centrifugal or diffuse	7(25.9%)	8(38.1%)	
Homogeneity of enhancement			<0.001***
Homogeneous	14(51.9%)	1(4.8%)	
Heterogeneous	13(48.1%)	20(95.2%)	
Perfusion defect^#^			<0.001***
Present	17(63.0%)	19(90.5%)	
Absent	10(37.0%)	2(9.5%)	

Data were numbers of tumors, with percentages in parentheses.

# Perfusion defect: localized non-enhancement.

PTC, papillary thyroid carcinoma; ATC, anaplastic thyroid carcinoma; SWE, shear wave elastography.

*** *p*<0.001, ** *p*<0.01, * *p*<0.05.

The SE showed significant differences between the two groups (*p*=0.009). Scores of 4 (57.1% vs. 33.3%) and 5 (9.5% vs. 0.0%) based on the SE score criteria were more common in the ATC group (**Figures 1C**, **2C**) than in the PTC group. No significant differences were noted in the E-mean between the two groups (*p*>0.05, **Figures 1D**, **2D**).

### 3.2 Analysis of CEUS

**Table 1** shows the CEUS findings. Centripetal enhancement was the dominant pattern of enhancement direction in PTCs (74.1%) and ATCs (61.9%; *p*=0.325). A total of 51.9% (14/27) of PTCs showed homogeneous enhancement, and no perfusion defects were observed in 63.0% (17/27) of the PTCs. Unlike PTCs (**Figure 1E**), most ATCs showed heterogeneous enhancement (95.2%, *p*<0.001) and appeared as perfusion defects (90.5%, *p*<0.001; **Figure 2E**). The TIC analysis parameters of the tumor parenchyma and muscle adjacent to the tumor were not significantly different between the two groups, except for MTT in the tumor parenchyma (*p*=0.017, **Supplementary Tables 1**, **2**). Compared with the TIC analysis parameters of the PTC group (**Table 2**), those of the ATC group had a shorter MTT but higher PI and slope (*p*<0.05, **Figures 1F**, **2F**). No significant differences were noted in the TTP, AUC, WiAUC, or WoAUC (*p*>0.05).

**Table 2:** TIC parameters between PTC and ATC groups.

TIC parameters	PTC (*x ± s*)	ATC (*x ± s*)	*P* value
PI	271.90 ± 270.36	873.81 ± 1062.58	0.001**
Slope	112.64 ± 106.13	902.41 ± 2204.16	0.009**
TTP	6.32 ± 4.08	6.50 ± 7.55	0.216
MTT	80.46 ± 65.51	59.19 ± 73.03	0.045*
AUC	28040.69 ± 36153.24	44209.43 ± 76170.15	0.893
WiAUC	1237.75 ± 1672.73	3319.70 ± 5203.38	0.112
WoAUC	26802.93 ± 34758.73	40887.60 ± 73069.75	0.843

TIC, time-intensity curve; PTC, papillary thyroid carcinoma; ATC, anaplastic thyroid carcinoma; PI, peak intensity; TTP, time to peak; MTT, mean transit time; AUC, area under the curve; WiAUC, wash-in area under the curve; WoAUC, wash-out area under the curve.

** *p*<0.01, * *p*<0.05.

### 3.3 Analysis of immunohistochemistry

**Figure 3:**
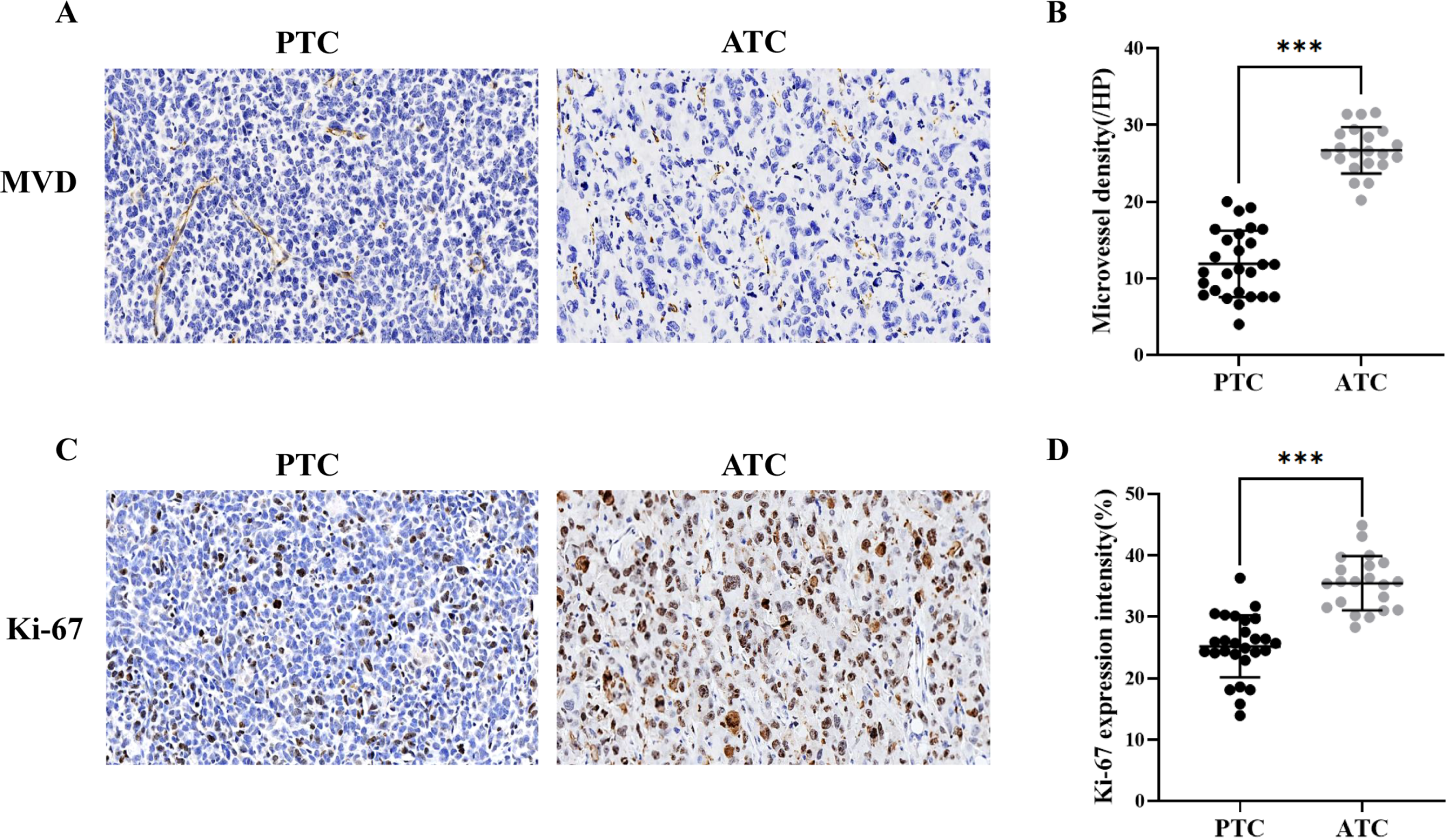
**(A)** Immunohistochemical analysis of the PTC group revealed an MVD of 14.6/HP (×20), and the ATC group revealed an MVD of 28.8/HP (×20). **(B)** The microvessel density was higher in the ATC group (n = 21) than in the PTC group (n = 27). **(C)** Immunohistochemical analysis reveals that the PTC group and the ATC group have a Ki-67 index of 26.1% (×20) and 32.4%, respectively (×20). **(D)** The expression intensity of Ki-67 in the ATC group (n = 21) was higher than in the PTC group (n = 27). ***p<0.001.

The ATC group exhibited a greater abundance of microvessels and higher expression intensity of Ki-67 than the PTC group (**Figure 3**). The counts of MVD were 26.70 ± 3.03 and 12.07 ± 4.23 in the ATC and PTC groups, respectively (*p*<0.001). The expression intensity of Ki-67 was 35.43 ± 4.41% and 25.44 ± 5.17% in the ATC and PTC groups, respectively (*p*<0.001).

### 3.4 Correlation between multimodal ultrasound and immunohistochemical markers

The correlations between multimodal ultrasound and IHC parameters are listed in **Supplementary Table 3**. The relationship between MVD and SE score, PI, slope, and the multimodal ultrasound model was statistically significant and positive (*p*<0.05). The inverse correlation between TTP, MTT, and MVD counts was statistically significant (*p*<0.05). Additionally, a positive correlation was observed between Ki-67 and PI, slope, WiAUC, and the multimodal ultrasound model (*p*<0.05). However, no such correlation was reported between the Adler grade, E-mean, AUC, WoAUC, and IHC parameters (*p*>0.05).

### 3.5 Diagnostic performance

**Figure 4:**
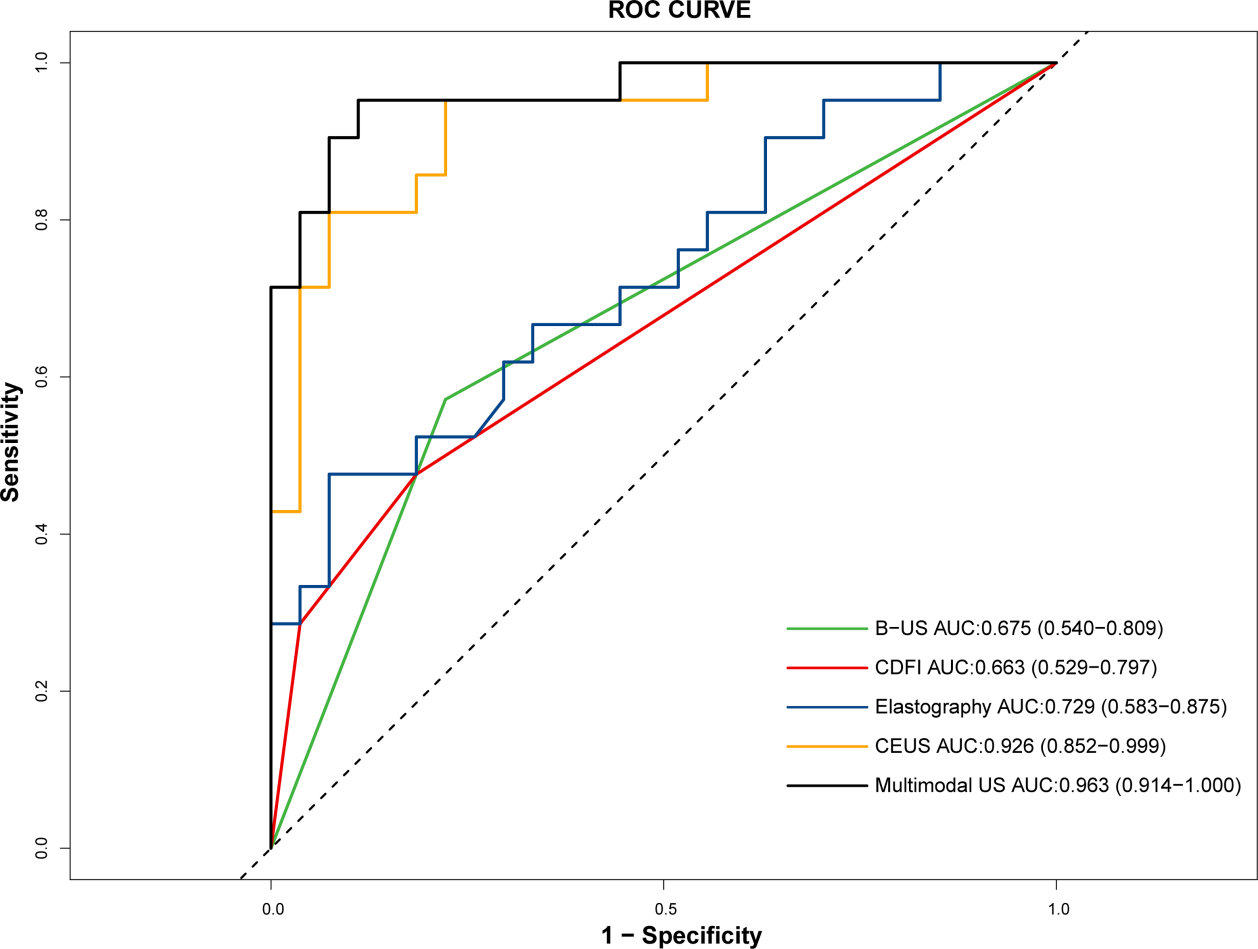
ROC curves of B-US, CDFI, elastography, CEUS, and multimodal US in distinguishing ATC (n = 21) from PTC (n = 27).

The diagnostic equation for distinguishing ATC from PTC using multimodal ultrasound was as follows: -8.944 + 4.029×Echogenicity (OR, 56.221 [95%CI: 2.264, 1396.210]; *p*=0.014) + 6.618×Perfusion defect (OR, 747.478 [95%CI: 7.422, 75278.607]; *p*=0.005) + 0.00689×PI (OR, 1.007 [95%CI: 1.002, 1.012]; *p*=0.006). In the formula, the variable “Echogenicity” is categorized into three levels: hypoechoic, isoechoic, and hyperechoic. These levels are assigned numerical codes as follows: = 0, = 1, and = 2. The binary factor “Perfusion defect” is given a value of 0 for absence and a value of 1 for presence. The diagnostic SEN, ACC, and NPV of the multimodal ultrasound model were higher than those of B-US, CDFI, Elastography and CEUS. The details are presented in **Table 3**. The AUC values for B-US, CDFI, elastography, CEUS, and multimodal US were 0.663, 0.729, 0.926, and 0.963, respectively (**Figure 4**). Statistically significant differences in the AUC values were found between CEUS and B-US (AUC, 0.926 vs. 0.675, *p*=0.004) and between multimodal US and B-US (AUC, 0.963 vs. 0.675, *p*<0.001).

**Table 3:** Diagnostic performance of B-US, CDFI, elastography, CEUS and multimodal US.

	AUC	SEN (%)	SPE (%)	ACC (%)	PPV (%)	NPV (%)	*P* value^#^
B-US	0.675	57.1	77.8	68.8	66.7	70.0	1.000
CDFI	0.663	47.6	81.5	66.7	66.7	66.7	0.877
Elastography	0.729	47.6	92.6	72.9	83.3	69.4	0.663
CEUS	0.926	81.0	92.6	87.5	89.5	86.2	0.004**
Multimodal US	0.963	95.2	88.9	91.7	87.0	96.0	<0.001***

# P value: P values of comparison of AUCs between B-US and other modal ultrasound calculated by the DeLong method.

B-US, B-mode ultrasound; CDFI, Color Doppler flow imaging; CEUS, contrast-enhanced ultrasound; AUC, area under the curve; SEN, sensitivity; SPE, specificity; ACC, accuracy; PPV, positive predictive value; NPV, negative predictive value.

*** *p*<0.001, ** *p*<0.01.

## 4 Discussion

This study evaluated the diagnostic performance of multimodal ultrasound, including B-US, CDFI, CEUS, and elastography, in distinguishing ATC from PTC and explored their correlation with markers of cancer aggressiveness. The rising prevalence of TC has facilitated the detection of ATC in microcarcinoma. Concurrently, the AS strategy for low-risk PTC has gained considerable acceptance, while the dedifferentiation of PTC to ATC during AS poses a significant risk associated with poor prognosis (8, 26). Therefore, exploring the role of ultrasound in differentiating PTC and ATC is imperative. To the best of our knowledge, this is the first study demonstrating the effectiveness of multimodal ultrasound in diagnosing ATC from PTC (AUC = 0.963). Moreover, ATC exhibited significantly higher levels of MVD and Ki-67, which are markers of TC aggressiveness, than did PTC. Quantified CEUS and SE showed significant correlations with MVD and Ki-67.In this study, microcalcification and echogenicity were significantly different between the ATC and PTC groups on B-US. Microcalcification was observed in 59.3% of PTC tumors compared to 19.0% of ATC tumors (*p*=0.005). Previous studies have shown that microcalcifications are common in PTC, likely caused by psammoma bodies (27, 28). Echogenicity emerged as the most sensitive indicator for differentiating ATC from PTC on B-US, but its SEN (57.1%) and SPE (77.8%), were suboptimal owing to overlapping features. These findings suggest that the diagnostic performance of B-US alone in distinguishing ATC from PTC remains limited.

In elastography, PTC tumors predominantly had SE scores of 2–4, whereas ATC tumors primarily had a score of 4–5, indicating higher stiffness in ATC. Increased stiffness in TC is influenced by factors such as fibrosis, calcification, and collagen content (29, 30). The high stiffness observed in ATC may be attributed to pronounced fibrosis and hyalinization, with spindle cells resembling fibroblasts or myofibroblasts histologically (31). However, SWE results showed no statistical difference, potentially due to differences in imaging principles (32). Technically, SE values, representing measured object stress, are more susceptible to applied pressure influence than SWE, which measures shear wave propagation speed and exhibits higher repeatability (33). In this study, the small size and fast heart rate of nude mice, along with their inability to hold their breath, caused significant interference from cardiac motion on tumors, impacting elasticity measurement quality. The superficial and protruding growth of subcutaneous tumors made uniform pressure application with the probe difficult, even with a gel pad, thus interfering with elasticity measurements. Moreover, ATC’s higher propensity for hemorrhage and necrosis, leading to greater heterogeneity, limited the pathological distinction of elasticity between ATC and PTC. Therefore, further studies in this regard are still required.

In our study, CDFI and CEUS were employed to assess TC vascularity. CDFI detected fast-flowing vessels (>1 cm/s) in larger vessels (>0.1 mm), providing macrovascular flow data (34), while CEUS identified microvessels under 40 μm, addressing microvascular assessment gaps (35). In this study, ATC exhibited a higher prevalence of Adler Grades 2 and 3 flows than did PTC, indicating a richer blood flow in ATC. CEUS frequently depicted heterogeneous enhancement and perfusion defects in ATC, contrasting with the homogeneous pattern observed in PTC. These differences are likely due to insufficient angiogenesis, hypoxia, and necrosis in rapidly growing malignant nodules (36). Our immunohistochemical results indicated that the MVD was higher in the ATC group, which suggested a greater number of new blood vessels in ATC. This led to the rapid and substantial influx of contrast agent into the vessels (37). Consequently, TIC showed lower MTT, along with higher PI and slope in ATC. Concurrently, owing to tumor necrosis in ATC, there is an absence of blood flow perfusion in certain areas. Therefore, there was no significant difference in AUC between the two groups, which represented the total blood volume.

The rich blood supply in ATC may contribute to its aggressive nature. We analyzed two key aggressiveness markers, Ki-67 and MVD (38, 39), and found significantly higher levels in ATC compared to PTC. To explore whether ultrasound features could predict aggressiveness, we examined the relationship between blood flow and Ki-67 expression, as reported in previous studies (40). However, our study found no significant correlation between CDFI and Ki-67, possibly due to the predominance of Adler Grade 1 flow in both the ATC and PTC groups. In contrast, higher Ki-67 expression was associated with a higher PI on CEUS TICs, suggesting a potential link between microperfusion and tumor aggressiveness. Consistent with earlier research (41, 42), MVD correlated positively with PI and slope and negatively with TTP and MTT. These findings indicate that CEUS TIC parameters may effectively reflect MVD and Ki-67 expression in TC, potentially serving as markers for malignancy and aggressiveness. Moreover, multimodal ultrasound was positively correlated with both MVD and Ki-67 expression, and the correlation was stronger than that of single-modality ultrasound. As discussed previously, perfusion defect and PI may be associated with rapid tumor growth and tumor angiogenesis. Therefore, multimodal ultrasound follow-up for comprehensive assessment of PTC progression during AS can indicate changes in the invasiveness of PTC.

To differentiate ATC from PTC, we evaluated the diagnostic performance of multimodal ultrasound imaging. CEUS outperformed other modalities, including B-US, CDFI, and elastography. Notably, the multimodal ultrasound model demonstrated optimal diagnostic efficacy, with an AUC of 0.963. It has been suggested that tumor size, the appearance of new lesions, and LNM should be closely monitored in patients with PTC undergoing AS (3, 12, 43). Lee, et al. (44) identified intratumoral vascularity as a risk factor for PTC progression. Our comprehensive analysis highlighted the importance of echogenicity, perfusion defects, and PI on TIC in distinguishing ATC from PTC. Therefore, we recommend regular multimodal ultrasound follow-ups, particularly CEUS, to dynamically detect microperfusion changes and assess PTC progression during AS.

The progression of PTC during AS is defined by tumor enlargement (increase in maximum diameter by ≥3 mm or ≥2 mm in at least two dimensions), ETE, or LNM. Active surgery is recommended only if the tumor enlarges to ≥13 mm, if ETE occurs, or if LNM is detected (12). However, B-US has limited sensitivity for diagnosing LNM. Therefore, we propose incorporating multimodal ultrasound, especially when tumor enlargement or LNM is suspected or when patients are undecided about transitioning to surgery. This approach could provide more comprehensive information for decision-making.

This study has several limitations. First, because of the extremely limited number of clinical ATC cases undergoing multimodal ultrasound imaging, we could not conduct retrospective cohort studies. Instead, we conducted preclinical investigations using human-derived cell lines. Second, the sample size of this study was relatively small, which might have limited its statistical power. Third, retrospective analysis of TIC curves may have introduced noise. Fourth, we utilized only CDFI without incorporating superb microvascular imaging when evaluating tumor vascularity. Both methods should be considered in future studies. Fifth, the concordance between the SE and SWE results is inconclusive, possibly due to the small sample size and uncontrollable breathing movements in the mice. Finally, limited by the model, this study failed to observe changes in the lymph nodes of PTC, which is one of the most concerning aspects of tumor progression during active surveillance. Further prospective, large-scale clinical studies are necessary to confirm the distinct multimodal ultrasonographic manifestations of ATC and PTC. Additionally, studies should also explore the prognostic and clinicopathological value of multimodal ultrasonography in AS of suspected progressive PTC.

## 5 Conclusions

This study demonstrated that, compared to PTC, ATC manifested more isoechoic, less microcalcification, higher SE scores, and higher microvascular perfusion as assessed by CEUS TIC analysis. Multimodal ultrasound, particularly CEUS, effectively differentiated ATC from PTC. Quantitative analysis of CEUS and SE revealed significant correlations with MVD and Ki-67 expression, both of which were elevated in patients with ATC. We propose that the follow-up strategy during AS of PTC should incorporate multimodal ultrasound, focusing on CEUS.

## Data Availability

The original contributions presented in the study are included in the article/**Supplementary Material**. Further inquiries can be directed to the corresponding author/s.

## References

[1] Zhu X Yao J Tian W. Microarray technology to investigate genes associated with papillary thyroid carcinoma. Mol Med Rep. (2015) 11:3729–33. doi: 10.3892/mmr.2015.3180 25586635

[2] Sebastian SO Gonzalez JM Paricio PP Perez JS Flores DP Madrona AP. Papillary thyroid carcinoma: prognostic index for survival including the histological variety. Arch Surgery. (2000) 135:272–7. doi: 10.1001/archsurg.135.3.272 10722027

[3] Miyauchi A Ito Y Fujishima M Miya A Onoda N Kihara M. Long-term outcomes of active surveillance and immediate surgery for adult patients with low-risk papillary thyroid microcarcinoma: 30-year experience. Thyroid: Off J Am Thyroid Assoc. (2023) 33:817–25. doi: 10.1089/thy.2023.0076 PMC10354707 37166389

[4] Sugitani I Ito Y Takeuchi D Nakayama H Masaki C Shindo H. Indications and strategy for active surveillance of adult low-risk papillary thyroid microcarcinoma: consensus statements from the Japan association of endocrine surgery task force on management for papillary thyroid microcarcinoma. Thyroid: Off J Am Thyroid Assoc. (2021) 31:183–92. doi: 10.1089/thy.2020.0330 PMC7891203 33023426

[5] Sugitani I. Active surveillance of low-risk papillary thyroid microcarcinoma. Best Pract Res Clin Endocrinol Metab. (2023) 37:101630. doi: 10.1016/j.beem.2022.101630 35256266

[6] Boumahdi S de Sauvage FJ. The great escape: tumour cell plasticity in resistance to targeted therapy. Nat Rev Drug Discov. (2020) 19:39–56. doi: 10.1038/s41573-019-0044-1 31601994

[7] Molinaro E Romei C Biagini A Sabini E Agate L Mazzeo S. Anaplastic thyroid carcinoma: from clinicopathology to genetics and advanced therapies. Nat Rev Endocrinology. (2017) 13:644–60. doi: 10.1038/nrendo.2017.76 28707679

[8] Luo H Xia X Kim GD Liu Y Xue Z Zhang L. Characterizing dedifferentiation of thyroid cancer by integrated analysis. Sci Adv. (2021) 7(31):eabf3657. doi: 10.1126/sciadv.abf3657 34321197 PMC8318367

[9] Bible KC Kebebew E Brierley J Brito JP Cabanillas ME Clark TJ. 2021 American thyroid association guidelines for management of patients with anaplastic thyroid cancer. Thyroid: Off J Am Thyroid Assoc. (2021) 31:337–86. doi: 10.1089/thy.2020.0944 PMC8349723 33728999

[10] Haddad RI Lydiatt WM Ball DW Busaidy NL Byrd D Callender G. Anaplastic thyroid carcinoma, version 2.2015. J Natl Compr Canc Netw. (2015) 13:1140–50. doi: 10.6004/jnccn.2015.0139 PMC4986600 26358798

[11] Tiedje V Stuschke M Weber F Dralle H Moss L Führer D. Anaplastic thyroid carcinoma: review of treatment protocols. Endocrine-related cancer. (2018) 25:R153–R61. doi: 10.1530/ERC-17-0435 29295821

[12] Lee JY Lee MK Lim HK Lee CY Sung JY Yoon JH. Standardized ultrasound evaluation for active surveillance of low-risk thyroid microcarcinoma in adults: 2024 korean society of thyroid radiology consensus statement. Korean J Radiol. (2024) 25:942–58. doi: 10.3348/kjr.2024.0871 PMC11524690 39473087

[13] Yu Qing D Jing B Bing W Song W Fei ZQ Kun Y. Differential diagnosis of pathological type of peripheral lung cancer with multimodal contrast-enhanced ultrasound. Ultrasound Med Biol. (2024) 50:1485–93. doi: 10.1016/j.ultrasmedbio.2024.05.017 39048469

[14] Guo J Jiang D Qian Y Yu J Gu Y-J Zhou Y-Q. Differential diagnosis of different types of solid focal liver lesions using two-dimensional shear wave elastography. World J Gastroenterol. (2022) 28:4716–25. doi: 10.3748/wjg.v28.i32.4716 PMC9476867 36157921

[15] Li G Ma S Zhang F Jia C Liu L Gao F. The predictive models based on multimodality ultrasonography for the differential diagnosis of thyroid nodules smaller than 10 mm. Br J Radiol. (2023) 96:20221120. doi: 10.1259/bjr.20221120 37427752 PMC10461269

[16] Melnik D Sahana J Corydon TJ Kopp S Nassef MZ Wehland M. Dexamethasone inhibits spheroid formation of thyroid cancer cells exposed to simulated microgravity. Cells. (2020) 9(2):367. doi: 10.3390/cells9020367 32033410 PMC7072698

[17] Gallo C Fragliasso V Donati B Torricelli F Tameni A Piana S. The bHLH transcription factor DEC1 promotes thyroid cancer aggressiveness by the interplay with NOTCH1. Cell Death Dis. (2018) 9:871. doi: 10.1038/s41419-018-0933-y 30158530 PMC6115386

[18] Adler DD Carson PL Rubin JM Quinn-Reid D. Doppler ultrasound color flow imaging in the study of breast cancer: preliminary findings. Ultrasound Med Biol. (1990) 16:553–9. doi: 10.1016/0301-5629(90)90020-D 2238263

[19] Itoh A Ueno E Tohno E Kamma H Takahashi H Shiina T. Breast disease: clinical application of US elastography for diagnosis. Radiology. (2006) 239:341–50. doi: 10.1148/radiol.2391041676 16484352

[20] Liu Y Liu H Qian C-L Lin M-S Li F-H. Utility of quantitative contrast-enhanced ultrasound for the prediction of extracapsular extension in papillary thyroid carcinoma. Sci Rep. (2017) 7:1472. doi: 10.1038/s41598-017-01650-2 28469180 PMC5431210

[21] Wan CF Du J Fang H Li FH Zhu JS Liu Q. Enhancement patterns and parameters of breast cancers at contrast-enhanced US: correlation with prognostic factors. Radiology. (2012) 262:450–9. doi: 10.1148/radiol.11110789 22282183

[22] Tang J Gui C Qiu S Wang M. The clinicopathological significance of Ki67 in papillary thyroid carcinoma: a suitable indicator? World J Surg Oncol. (2018) 16:100. doi: 10.1186/s12957-018-1384-8 29855303 PMC5984434

[23] Weidner N Semple JP Welch WR Folkman J. Tumor angiogenesis and metastasis–correlation in invasive breast carcinoma. New Engl J Med. (1991) 324:1–8. doi: 10.1056/NEJM199101033240101 1701519

[24] Kim JJ Kim JH Koo JK Choi YJ Ko SY Choe WH. The Refit model for end-stage liver disease-Na is not a better predictor of mortality than the Refit model for end-stage liver disease in patients with cirrhosis and ascites. Clin Mol Hepatol. (2014) 20:47–55. doi: 10.3350/cmh.2014.20.1.47 24757658 PMC3992329

[25] Liu W Wang S Xia X Guo M. A proposed heterogeneous ensemble algorithm model for predicting central lymph node metastasis in papillary thyroid cancer. Int J Gen Med. (2022) 15:4717–32. doi: 10.2147/IJGM.S365725 PMC9091701 35571287

[26] Landa I Ibrahimpasic T Boucai L Sinha R Knauf JA Shah RH. Genomic and transcriptomic hallmarks of poorly differentiated and anaplastic thyroid cancers. J Clin Invest. (2016) 126:1052–66. doi: 10.1172/JCI85271 PMC4767360 26878173

[27] Li X Zhou W Zhan W. Clinical and ultrasonographic features of medullary thyroid microcarcinomas compared with papillary thyroid microcarcinomas: a retrospective analysis. BMC Med imaging. (2020) 20:49. doi: 10.1186/s12880-020-00444-9 32410587 PMC7227110

[28] Kim BK Lee EM Kim JH Oak SY Kwon SK Choi YS. Relationship between ultrasonographic and pathologic calcification patterns in papillary thyroid cancer. Medicine. (2018) 97:e12675. doi: 10.1097/MD.0000000000012675 30313060 PMC6203561

[29] Rago T Scutari M Loiacono V Santini F Tonacchera M Torregrossa L. Low elasticity of thyroid nodules on ultrasound elastography is correlated with Malignancy, degree of fibrosis, and high expression of galectin-3 and fibronectin-1. Thyroid: Off J Am Thyroid Assoc. (2017) 27:103–10. doi: 10.1089/thy.2016.0341 27809694

[30] Yi L Qiong W Yan W Youben F Bing H. Correlation between ultrasound elastography and histologic characteristics of papillary thyroid carcinoma. Sci Rep. (2017) 7:45042. doi: 10.1038/srep45042 28327620 PMC5361199

[31] Ragazzi M Ciarrocchi A Sancisi V Gandolfi G Bisagni A Piana S. Update on anaplastic thyroid carcinoma: morphological, molecular, and genetic features of the most aggressive thyroid cancer. Int J Endocrinol. (2014) 2014:1–13. doi: 10.1155/2014/790834 PMC4158294 25214840

[32] Huang S Ye X Yang K Tian H Ding Z Chen J. The significance of dual-mode elastography in the diagnosis of breast lesions by physicians with different levels of experience. Quant Imaging Med Surg. (2022) 12:1438–49. doi: 10.21037/qims-21-636 PMC8739147 35111637

[33] Gennisson JL Deffieux T Fink M Tanter M. Ultrasound elastography: principles and techniques. Diagn Interv Imaging. (2013) 94:487–95. doi: 10.1016/j.diii.2013.01.022 23619292

[34] Zhang G Lei YM Li N Yu J Jiang XY Yu MH. Ultrasound super-resolution imaging for differential diagnosis of breast masses. Front Oncol. (2022) 12:1049991. doi: 10.3389/fonc.2022.1049991 36408165 PMC9669901

[35] Niu X Jiang W Zhang X Ding Z Xue H Wang Z. Comparison of contrast-enhanced ultrasound and positron emission tomography/computed tomography (PET/CT) in lymphoma. Med Sci monitor: Int Med J Exp Clin Res. (2018) 24:5558–65. doi: 10.12659/MSM.908849 PMC6098669 30095086

[36] Metz S Daldrup-Unk HE Richter T Rath C Ebert W Settles M. Detection and quantification of breast tumor necrosis with MR imaging: value of the necrosis-avid contrast agent Gadophrin-3. Acad radiology. (2003) 10:484–90. doi: 10.1016/S1076-6332(03)80056-9 12755535

[37] Duan S Zhang Y Xu S Jiang P Qi Q. Contrast-enhanced ultrasound parameters and D-dimer: new prognostic parameters for diffuse large B-cell lymphoma. Cancer Manage Res. (2022) 14:2535–44. doi: 10.2147/CMAR.S326173 PMC9426867 36051181

[38] Ward Y Lake R Martin PL Killian K Salerno P Wang T. CD97 amplifies LPA receptor signaling and promotes thyroid cancer progression in a mouse model. Oncogene. (2013) 32:2726–38. doi: 10.1038/onc.2012.301 PMC7561260 22797060

[39] Skuletic V Radosavljevic GD Pantic J Markovic BS Jovanovic I Jankovic N. Angiogenic and lymphangiogenic profiles in histological variants of papillary thyroid carcinoma. Pol Arch Intern Med. (2017) 127:429–37. doi: 10.20452/pamw.3999 28425432

[40] Cheng C Zhao H Tian W Hu C Zhao H. Predicting the expression level of Ki-67 in breast cancer using multi-modal ultrasound parameters. BMC Med imaging. (2021) 21:150. doi: 10.1186/s12880-021-00684-3 34656085 PMC8520259

[41] Li M-H Li W-W He L Li J-F Zhang S-Y. Quantitative evaluation of colorectal tumour vasculature using contrast-enhanced ultrasound: Correlation with angiogenesis and prognostic significance. World J Gastrointest Surg. (2023) 15:2052–62. doi: 10.4240/wjgs.v15.i9.2052 PMC10600759 37901730

[42] Li X Li Y Zhu Y Fu L Liu P. Association between enhancement patterns and parameters of contrast-enhanced ultrasound and microvessel distribution in breast cancer. Oncol Lett. (2018) 15:5643–9. doi: 10.3892/ol.2018.8078 PMC5844090 29556301

[43] Ito Y Miyauchi A Fujishima M Noda T Sano T Sasaki T. Thyroid-stimulating hormone, age, and tumor size are risk factors for progression during active surveillance of low-risk papillary thyroid microcarcinoma in adults. World J surgery. (2023) 47:392–401. doi: 10.1007/s00268-022-06770-z PMC9803751 36182976

[44] Lee JY Kim J-H Kim YK Lee CY Lee EK Moon JH. US predictors of papillary thyroid microcarcinoma progression at active surveillance. Radiology. (2023) 309:e230006. doi: 10.1148/radiol.230006 37906009

