# Supplementary material for: Multimodal ultrasound: a non-invasive method for identifying dedifferentiation of papillary thyroid carcinoma during active surveillance
Source: Front Oncol. 2025 Feb 19;15:1545407. doi: 10.3389/fonc.2025.1545407 (PMC11879784; doi:10.3389/fonc.2025.1545407)
Supplement: Supplementary file 1 [file DataSheet1.docx]

Supplementary Material

# Supplementary Figures and Tables

## Supplementary Figures


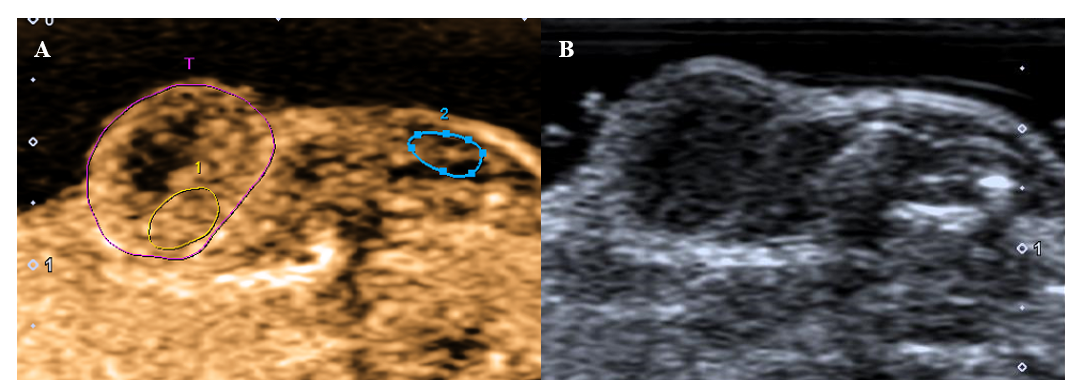


**Supplementary Figure 1.** Schematic of various ROI outlines in CEUS images. Real-time synchronous images of CEUS (A) and B-US (B). Purple: the entire tumor. Yellow: tumor parenchyma (exclude the necrotic areas). Blue: the muscle next to the tumor.

## Supplementary Tables

**Supplementary Table 1.** TIC parameters of tumor parenchyma between ATC and PTC groups

| TIC parameters | PTC (*x ± s*) | ATC (*x ± s*) | *P* value |
| --- | --- | --- | --- |
| TTP | 4.80±3.66 | 5.89±7.47 | 0.596 |
| MTT | 59.26±71.72 | 43.33±69.03 | 0.017* |
| PI | 722.17±755.94 | 3132.73±9139.99 | 0.209 |
| Slope | 425.52±579.15 | 6148.92±2204.16 | 0.209 |
| AUC | 68735.54±115827.40 | 46600.43±73015.12 | 0.486 |
| WiAUC | 2566.12±3964.01 | 3310.74±5354.24 | 0.499 |
| WoAUC | 66189.45±112211.56 | 43289.68±69104.44 | 0.701 |

TIC = time-intensity curve, PTC = papillary thyroid carcinoma, ATC = anaplastic thyroid carcinoma, PI = peak intensity, TTP = time to peak, MTT = mean transit time, AUC = area under the curve, WiAUC = wash-in area under the curve, WoAUC = wash-out area under the curve.

**P* < 0.05

**Supplementary Table 2.** TIC parameters of the muscle next to the tumor between ATC and PTC groups

| TIC parameters | PTC (*x ± s*) | ATC (*x ± s*) | *P* value |
| --- | --- | --- | --- |
| TTP | 21.4±34.41 | 12.49±13.58 | 0.339 |
| MTT | 65.19±65.46 | 42.84±59.18 | 0.140 |
| PI | 228.50±200.00 | 416.09±379.68 | 0.055 |
| Slope | 66.01±101.39 | 152.41±193.78 | 0.066 |
| AUC | 16847.74±13824.87 | 21458.32±28890.45 | 0.959 |
| WiAUC | 2649.87±4822.96 | 3222.10±5084.20 | 0.992 |
| WoAUC | 14207.87±13562.22 | 18236.21±25658.80 | 0.959 |

TIC = time-intensity curve, PTC = papillary thyroid carcinoma, ATC = anaplastic thyroid carcinoma, PI = peak intensity, TTP = time to peak, MTT = mean transit time, AUC = area under the curve, WiAUC = wash-in area under the curve, WoAUC = wash-out area under the curve.

**Supplementary Table 3.** The correlation of multimodal ultrasound with MVD and the expression intensity of Ki-67

|  | MVD | | Ki-67 | |
| --- | --- | --- | --- | --- |
|  | *r* | *P* value | *r* | *P* value |
| Adler grade | 0.253 | 0.083 | -0.226 | 0.122 |
| SE score | 0.457 | 0.001** | 0.102 | 0.491 |
| Emean | -0.029 | 0.843 | -0.069 | 0.639 |
| TTP | -0.360 | 0.012* | -0.068 | 0.647 |
| MTT | -0.310 | 0.032* | -0.106 | 0.475 |
| PI | 0.378 | 0.008** | 0.449 | 0.001** |
| Slope | 0.440 | 0.002** | 0.317 | 0.028* |
| AUC | -0.009 | 0.954 | 0.163 | 0.269 |
| WiAUC | 0.013 | 0.929 | 0.309 | 0.033* |
| WoAUC | -0.04 | 0.787 | 0.106 | 0.471 |
| Multimodal ultrasound model | 0.725 | <0.001*** | 0.536 | <0.001*** |

MVD = microvessel density, PI = peak intensity, TTP = time to peak, MTT = mean transit time, AUC = area under the curve, WiAUC = wash-in area under the curve, WoAUC = wash-out area under the curve.

****P* < 0.001 ,***P* < 0.01, **P* < 0.05
